# Neuroprotective and Neurorestorative Processes after Spinal Cord Injury: The Case of the Bulbospinal Respiratory Neurons

**DOI:** 10.1155/2016/7692602

**Published:** 2016-08-03

**Authors:** Anne Kastner, Valéry Matarazzo

**Affiliations:** ^1^PPSN EA 4674, Aix-Marseille Université, 13013 Marseille, France; ^2^INMED UMR 901, INSERM, Aix-Marseille Université, 13273 Marseille, France

## Abstract

High cervical spinal cord injuries interrupt the bulbospinal respiratory pathways projecting to the cervical phrenic motoneurons resulting in important respiratory defects. In the case of a lateralized injury that maintains the respiratory drive on the opposite side, a partial recovery of the ipsilateral respiratory function occurs spontaneously over time, as observed in animal models. The rodent respiratory system is therefore a relevant model to investigate the neuroplastic and neuroprotective mechanisms that will trigger such phrenic motoneurons reactivation by supraspinal pathways. Since part of this recovery is dependent on the damaged side of the spinal cord, the present review highlights our current understanding of the anatomical neuroplasticity processes that are developed by the surviving damaged bulbospinal neurons, notably axonal sprouting and rerouting. Such anatomical neuroplasticity relies also on coordinated molecular mechanisms at the level of the axotomized bulbospinal neurons that will promote both neuroprotection and axon growth.

## 1. Introduction

Traumatic spinal cord injuries (SCI) often result in severe lifelong motor and autonomic disabilities, generally due to the interruption of the supraspinal descending pathways. Because axon fibers cannot spontaneously regenerate in the central nervous system (CNS), numerous studies are aimed to develop restorative treatments designed to repair the damaged pathways. The functional benefits resulting from some strategies applied in animals (as olfactory ensheathing cells transplantation or growth-promoting channels bridging the injury) are quite optimistic for putative translation in humans [1]. However, the functional benefit of these interventions may rely on the intrinsic features of the damaged neurons, such as their ability to react to the injury, to survive, and to sustain neuroplastic processes. Initial studies focused on corticospinal locomotor pathways allow putting forward the principle of post-SCI pathway reorganization [2]. This model however suffers from the rather poor growth-competence of these corticospinal neurons following SCI by comparison to other supraspinal neuronal populations, even in presence of growth-promoting strategies (see below).

**Figure 1:**
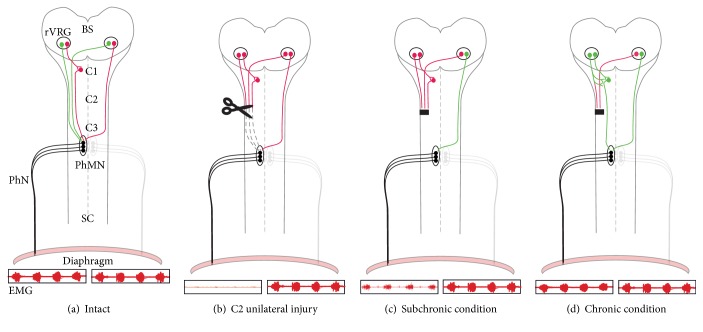
Diagram showing the spontaneous neural plasticity of the respiratory drive after cervical spinal cord injury. Green color represents active neural network. Red color represents silent or inactive neuronal network. (a, b) Unilateral injury leads to inactivation of ipsilateral premotoneurons from the brainstem or spinal cord (C1 interneurons). (c) In subchronic condition, the neurorestorative pathways include activation of CPP (contralateral premotoneurons in subchronic condition). (d) In chronic rerouting of unilateral premotoneurons through collateral connection to C1 interneurons. These last interneurons relay to phrenic motoneurons. BS: brainstem; C1–C3: cervical levels; EMG: electromyogram of hemidiaphragm SC: spinal cord; PMN: phrenic motoneuron; PN: phrenic nerve.

Beside locomotion, a devastating effect of cervical SCI can be breathing impairments. After cervical trauma in humans respiratory failure is the principal cause of mortality and morbidity and mechanical ventilator assistance is often required to compensate respiratory insufficiency [3–5]. The major inspiratory muscle in mammals is the diaphragm which is innervated by phrenic motoneurons (PhMN) located into the ventral horn of the cervical cord (C3–C6) [6]. These PhMN receive supraspinal projections from respiratory premotoneurons forming a column of cells in the ventrolateral medulla, the rostral ventral respiratory group (rVRG), a region just rostral to the obex [7, 8] (Figure 1(a)). These rVRG premotoneurons discharge during the inspiration in phase with the phrenic burst. They receive inputs from the pre-Bötzinger complex considered as being the main inspiratory rhythm generator [5, 7]. The descending bulbospinal respiratory pathways project not only to the phrenic nuclei but also to the thoracic motoneurons controlling intercostals muscles. In rodents, anterograde labeling by injections within one rVRG shows that labeled respiratory pathways originating from one medulla side project bilaterally in the SC, but still with predominance of ipsilateral (ipsi) projections [9, 10]. This feature is also confirmed by the fact that unilateral injection of a retrograde tracer into the phrenic area leads to bilateral labeling of the rVRGs [11]. Another feature of these respiratory descending pathways is that they can also project to the contralateral phrenic nucleus by crossing the spinal midline at the C3–C6 level [11, 12]. These “crossed phrenic pathways” (CPP) remain generally latent but they can be activated by respiratory stress conditions (Figure 1(a)). Although respiratory spinal projections to the phrenic nucleus are mainly monosynaptic, the PhMN may also receive inputs from relay pathways via spinal respiratory interneurons (SRI) located at the level of the phrenic nuclei or at the C1-C2 level [6, 13] (Figure 1(a)).

The canonical experimental model to study the effect of high cervical SCI on the respiratory system is a unilateral section at the C2 level [3]. Such C2 injury damages all ipsi respiratory spinal projections and consequently interrupts phrenic and diaphragmatic activities of the damaged side so that the respiratory function relies only on the opposite hemidiaphragm (Figure 1(b)). Another experimental model, which may better reflect the physiopathology of trauma injury in human, is the cervical unilateral contusion model [3]. It leads to a substantial impairment of ipsi hemidiaphragm activity but generally not as important as for cervical sections [14]. In C2 hemisected animals, a well-studied neuroplasticity process is the “crossed phrenic phenomenon”: it refers to the partial ipsi phrenic nerve (PhN) recovery evoked by some respiratory stress as classically by a transection of the contralateral PhN [12]. This process relies on the rapid activation of the normally silent CPP innervating the ipsi phrenic nucleus (see above). Such “crossed phrenic phenomenon” is not evoked by the C2 hemisection itself, since the respiratory stress is limited by various compensatory mechanisms involving the spared respiratory pathways. However, even in absence of additional respiratory stress, CPP-dependent PhN recovery appears spontaneously few days following the C2 hemisection [15] (Figure 1(c)). It suggests that SCI may initiate neuroplasticity processes leading to a shift from latent toward active CPP. More generally, extensive anatomical neuroplasticity processes may operate during the weeks and months following high cervical SCI to reinnervate and reactivate the PhMN leading to a respiratory functional improvement. Thus, in cervical injured patients who require ventilatory assistance, a long-term diaphragmatic recovery can sometimes occur [3]. Similarly, in rats with C2 unilateral SCI, several studies reveal a progressive spontaneous recovery of the ipsi PhN activity in relation to the postlesion time, reaching around 40% from the initial value several months after lesion [16–18]. Although most studies have been focused on the contralateral spared CPP pathways, they may become less essential after long-term SCI [16, 18], suggesting that additional neuroplastic mechanisms may occur in chronic conditions. The aim of this present review is therefore to emphasize the postlesion neuroplastic mechanisms related to the damaged respiratory bulbospinal neurons and pathways: axon sprouting and pathways reorganization (Section 2), molecular cell signaling promoting both neuron survival and growth-competence (Section 3).

## 2. Part I: Plasticity Processes of the Damaged Bulbospinal Pathways and Their Involvement in Respiratory Recovery

### 2.1. Regenerative Sprouting and Relevance of the Respiratory System

#### 2.1.1. Influence of the Proximity to the Injury Site on Regenerative Sprouting

After a partial SCI, the axotomized axons distal to the injury degenerate completely whereas their proximal axonal elements usually survive. In the Mammalian CNS, by contrast to the PNS, growth of these injured axons is however very limited due to the inhibitory environment of the CNS. Thus, in absence of growth-promoting interventions, damaged axons cannot regenerate spontaneously throughout the spinal scar to reinnervate and target their motoneurons. Nevertheless, previous studies using the model of the corticospinal tract injury have described important axonal sprouting and rerouting toward the grey matter [2, 19]. Following a dorsal SCI, Steward et al. [20] have shown that a few dorsal axotomized corticospinal terminals (1%) can even regrow toward the ventral SC and thus bypass the injury site. However, in quite similar rodent models (lateral or dorsal SCI), other studies did not detect any corticospinal axon outgrowth in absence of growth-promoting therapeutic interventions [21, 22], so that the experimental conditions that permit or not spontaneous sprouting of the corticospinal fibers are not yet clearly defined. After SCI, the rather poor growth potential of corticospinal fibers has also been shown in various permissive conditions which in counterpart allow the regeneration of other supraspinal neurons [23–27].

One critical parameter that determinates the growth and regenerative potential of the injured axons is the distance between the site of axotomy and the cell body [28]. Thus, for a given SCI site, cell bodies of the corticospinal tract are more distant from the injury than brainstem bulbospinal neurons (among which respiratory neurons). It may explain that only the latter are able to regenerate within a growth permissive guidance channel (as a peripheral nerve graft) inserted at the level of the SCI site, whereas neurons of the motor cortex failed to regenerate their axons [26, 29–32]. The link between the regenerative potential and the cell body distance has been directly demonstrated for propriospinal interneurons, with the number of regenerated cells decreasing progressively with increasing distance from the lesion site [31]. The same conclusion can be drawn from the observation that brainstem neurons are able to regenerate into peripheral nerve transplants after cervical but not thoracic SCI [33]. Thus, after cervical SCI and even in absence of growth-promoting interventions, the sprouting of brainstem bulbospinal neurons (as in the case of the respiratory and locomotor systems) may be favored by the short distance between the cell body and the injury site (a few mm in the rat). Thus, in the case of the locomotor system, the bulbospinal fibers (originating from the Gigantocellularis nucleus) are able to develop collaterals within the grey matter above a cervical lateral hemisection [34], whereas such sprouting was not detected after a thoracic hemisection [35]. Thus, due to their very close proximity from a high cervical SCI in comparison to other brainstem neurons, the respiratory bulbospinal neurons may be a particular relevant model to analyze such spontaneous anatomical plasticity. Moreover, even if axonal regeneration may be improved by some experimental conditions, its success and benefit may be compromised by the long distance required for reinnervation of the target motoneurons, especially in the case of cervical injuries. For the respiratory system, however, a high cervical SCI is particularly close to the main target sites of reinnervation, the PhMN (C3–C6 in rats and man).

#### 2.1.2. Regenerative Processes of the Axotomized Respiratory Pathways

Previous studies have demonstrated the possibility for the bulbospinal respiratory fibers to regrow within a growth permissive nerve graft inserted near the lesion site that contains the damaged descending respiratory tract [30, 36]. In these two studies the presence within the nerve graft of efferent respiratory axons was confirmed by the recording from teased fibers of unitary discharges corresponding to respiratory bursts and also by the labeling of rVRG neurons after injection of a retrograde tracer in the distal part of the nerve graft. Hence, when the nerve graft was used to bridge the injury (in combination with chondroïtinase treatment in order to digest the inhibitory extracellular matrix), the regeneration of respiratory neurons was accompanied by a remarkable diaphragm recovery. This recovery was completely eliminated by transection of the graft bridge [37], indicating that it relies on axon regeneration within the nerve channel. These studies altogether indicate that respiratory neurons have a good intrinsic ability to grow and regenerate although the CNS environment may represent a limiting factor. Moreover, even in absence of reparative strategies, the axotomized respiratory neurons identified by retrograde labeling exhibit a rather good survival after a chronic C2 SCI [38].

In order to assess the possibility of spontaneous sprouting in these axotomized respiratory neurons, we identified the bulbospinal respiratory fibers by injecting an anterograde marker in the area of the inspiratory center (rVRG). As observed in previous studies [10, 39, 40], the labeled bulbospinal respiratory fibers were located principally in the ventrolateral sector of the SC with some collaterals decussating to laminae VII and X [9] (Figure 1(a)). In rats with a lateralized C2 injury, the number of respiratory fibers innervating the ipsi phrenic nucleus was, as expected, much reduced (reaching 5–10% from the initial value), even after long delay, showing that the damaged ipsi respiratory fibers did not spontaneously regenerate [9]. In these C2 injured animals, we found also a reduced number of respiratory-putative fibers in the lateral ipsi C1 compartment due to putatively axonal retraction rather than to neurodegeneration, in correlation with the white matter shrinkage around the lesion site. However, despite this process of axonal retraction in the ipsi C1 ventrolateral area, collateral arborization of the respiratory bulbospinal fibers within the C1 ventral grey matter appeared increased in chronically injured animals [9] (Figure 1(d)), as observed also more recently for bulbospinal fibers originating from the reticular formation [34]. These data are also in agreement with the higher level of axon growth marker GAP-43 detected in the injured spinal cord at C1 level [9] and may indicate an induction of axonal sprouting in C2 injured animals. Moreover, in rats with a chronic C2 lateral SCI, we could observe, just rostrally to the glial scar, numerous fibers (5%) that turn perpendicularly toward the grey matter and that were thus rerouted toward the medial SC [9]. Thus, axotomy of bulbospinal respiratory pathways by a lateral C2 SCI induces regenerative processes as axonal sprouting and rerouting. However, although some fibers may be able to bypass the glial scar by the medial side, our anterograde and retrograde tracing experiments did not reveal any detectable reinnervation of the phrenic area by these injured fibers. In addition to axon sprouting, other neuroplasticity processes may therefore occur to account for the spontaneous long-term phrenic and diaphragmatic reactivation.

### 2.2. Rewiring Processes of Damaged Bulbospinal Pathways and Their Functional Incidence

#### 2.2.1. Spared versus Damaged Restorative Pathways

The recovery of the respiratory function after a cervical C2 hemisection has classically been attributed to the activation of spared contralateral pathways that cross the spinal midline below the C2 lesion to innervate the ipsi PhMN. These CPP pathways are latent under normal respiratory conditions but became progressively recruited following SCI, leading to the restoration of the inspiratory drive to the ipsi phrenic nucleus [15, 17, 41, 42]. Thus, in rats, one week after a lateral C2 SCI (that suppressed all ipsi phrenic drive in acute conditions), the recovered ipsi PhN activity (20% from the contralateral PhN) was indeed completely inactivated immediately after a secondary contralateral C1 hemisection so that animal survival was compromised in absence of ventilator assistance [15]. This supplementary contralateral C1 hemisection suppressed indeed all contralateral respiratory drive, including the CPP innervating the ipsi phrenic nucleus. However, in conditions of chronic complete hemisection, some studies show either no ipsi PhN activity [37, 43] or only a modest recovery [44] so that the effective contribution of CPP pathways remains controversial. Two months after a C2 hemisection in rats, Li et al. [43] observed that ipsi PhN remains inactive if the hemisection was complete but was activated in the case of ventromedial tissue sparing. Thus, in this latter case, neural substrate other than CPP pathways may be engaged in spontaneous functional improvement. In line with this assumption, we show that, after a chronic lateral (partial) C2 SCI, the recovery of ipsi PhN activity was almost independent of the integrity of the contralateral side [18]. These results show that neuroplasticity processes following SCI may also engage pathways present in the damaged ipsi side that may bypass the lesion through the medial SC region. This raises the question of the putative reorganization of the injured pathways and their contribution to respiratory recovery.

#### 2.2.2. Respiratory Propriospinal Neurons after a C2 SCI

While little is known about neuronal substrate of post-SCI structural reactive plasticity, a first study on the model of the corticospinal tract reported that propriospinal interneurons are involved in rewiring processes after thoracic SCI and may play crucial role in postinjury recovery of function [2]. In the respiratory system, although direct monosynaptic projections from the rVRG neurons to PhMN may dominate, the presence of propriospinal respiratory neurons (SRI) in the high cervical SC has also been demonstrated by several electrophysiological and anatomical studies in intact rodents [40, 45–47]. Thus, cross correlation analysis revealed the presence of monosynaptic connections between the rVRG and C1/C2 inspiratory units and between these C1/C2 propriospinal neurons and the PhMN [47, 48]. These data suggest that the PhMN may also be innervated by an indirect polysynaptic pathway involving C1/C2 SRI. Using neurobiotin intracellular anterograde tracer, Lipski et al. [40] indeed observed that SRI had long propriospinal projections with a short collateral which arborized in the region of the PhMN. The anatomical connection between these SRI and the PhMN has been confirmed by other studies using retrograde transsynaptic tracer [49–51]. Indeed, pseudorabies virus injected into the PhN permits us to label second-order neurons (connected to the PhMN pool) at the level of the phrenic nucleus but also small neurons in the upper cervical SC (C1–C3). These SRI are principally located in the intermediate and medial part of the SC, in lamina X and VII, and are synaptically coupled to both central respiratory neurons (within the rVRG) and PhMN [50]. Moreover, using a voltage-sensitive dye on isolated brainstem-SC preparations from neonatal rats, respiratory-related activity can be visualized in C1-C2 spinal neurons that may therefore correspond to SRI [46]. SRI may represent a heterogeneous population that may mediate descending supraspinal input to PhMN but that may also integrate numerous other neuronal circuits and that may coordinate bilateral PhN activity and also PhN/intercostals nerve activity [13, 50].

These different features concerning SRI raise the question of their putative contribution to restorative processes in the respiratory system. In rats with complete hemisection (two weeks after SCI) that will damage all ipsi pathways, an apparent reduction in rVRG projections to SRI and also of the SRI projections to the PhMN has been reported (even at 3 months after SCI) [13, 50]. A complete hemisection will indeed interrupt both the direct and indirect pathways to respiratory PhMN, so that only CPP pathways (direct or indirect) may still be able to innervate the ipsi PhMN pool. Indirect pathways may rather transit principally in the medial compartment of the SC where the SRI are located so that their contribution to respiratory recovery may be favored by a C2 SCI injury with minimal ventromedial sparing. Such ventromedial sparing (by comparison with complete C2 hemisection) can indeed improve respiratory recovery [9, 43, 52], putting forward the contribution of medial transiting respiratory pathways (see next paragraph). The putative involvement of SRI to respiratory restorative pathways was therefore analyzed in rats with a SCI that may leave intact pathways transiting through the medial part of the SC [9]. Anterograde/retrograde dual tracing revealed bulbospinal fibers originating from the rVRG area (labeled by an anterograde tracer) that form close appositions to C1 ipsi propriospinal neurons projecting to C4 ventral horn (labeled by a retrograde tracer) (Figure 1(a)). We found that the number of these contacts was enhanced in animals with chronic lateral C2 SCI. This suggests that the injured axons that develop new collaterals toward the grey matter may form new connections to C1 propriospinal neurons in a way that the injured respiratory pathways may bypass the lateral injury via new indirect relay pathways that may reinnervate the ipsi PhMN (Figure 1(d)) [9]. More recently, the possibility for injured bulbospinal fibers to form such new relay pathways toward cervical motoneurons was also attested for the locomotor neurons originating from the reticular Gigantocellularis nucleus [34]. In this latter study, the presence of excitatory V-GLUT-positive varicosities on their terminals suggests the synaptic integration of these regrowing bulbospinal fibers [34]. In addition, this study and ours show an increased number of propriospinal neurons and fibers that bypass the injury and project beneath the injury site (Figure 1(d)). Accordingly, these newly engaged propriospinal neurons are predominantly located laterally (above the lateral injury) so that they may putatively correspond to injured interneurons that succeed in bypassing the injury by the medial part [9]. Thus, for both respiratory and locomotor bulbospinal pathways, the cervical motoneurons could be reinnervated by detour pathways formed by the connection of injured axons to propriospinal neurons that will thus bridge the injury. Such enhanced recruitment of propriospinal neurons bypassing the scar has also previously been demonstrated after a chronic thoracic hemisection [53].

#### 2.2.3. Functional Outcomes

In chronically C2 hemisected animals with ventromedial tissue sparing, respiratory recovery has been shown to be triggered principally by ipsi pathways that bypass the chronic C2 SCI by the medial region rather than by CPP reinforcement [18]. The recovered PhN activity was indeed maintained after a secondary section at C1/C2 on both sides that only spared the ipsi medial part [18], so that the animals could survive without ventilator assistance during the following hours. The contribution of these medial bypassing pathways is also suggested by the twice higher diaphragmatic recovery in animals with ventromedial sparing in comparison to rats with complete hemisection [9] and by their greater tidal volume during hypercapnic challenge [52]. Moreover, in chronically hemisected rats, a recovered PhN activity was detected only in the case of ventromedial tissue sparing [43]. Although the anatomical basis of such ventromedial-dependent restorative activity has not been clearly assessed, one possibility is that it may engage preexisting direct medial pathways (spared by a lateral SCI) rather than indirect relay pathways. The presence of these direct descending respiratory projections within the ventromedial spinal tissue has been shown by anterograde labeling [9, 10], although they are less abundant than the ventrolateral pathways. Still, these medial pathways are not efficient enough to trigger any PhN activity after an acute or subchronic (1 week) lateral C2 SCI sparing the medial spinal compartment [15]. It seems therefore rather unlikely that they could be sufficiently reinforced after a longer time-lag to be able to sustain by themselves the observed PhN recovery. Respiratory recovery may therefore more likely rely on respiratory pathways remodeling, as, for instance, with propriospinal respiratory neurons within the ventromedial compartment (Figure 1(d)).

Consistently with a greater functional involvement of such polysynaptic relay pathways through SRI, electrophysiological data from rats 12 W after C2 SCI showed a delayed activation of ipsi versus contralateral PhMN, indicating a prolonged conduction time to ipsi PhMN consecutively to postinjury plasticity processes [41, 54–56]. Moreover, in the case of respiratory recovery by a nerve graft bridging the C2 SCI, transection of the graft bridge initially led to an unusual increase in overall tonic diaphragm electromyographic activity (which does not occur after C2 hemisection alone) [37]. Due to the fact that the pattern of this activity was reminiscent of SRI recordings showing repeated transient increases in tonic spiking frequencies during inspiration, Alilain et al. suggest it could be attributed to the recruitment of such SRI that may play an important role in remodeling of the spinal respiratory network during the regenerative process.

While C2 hemisection is still the classical model to study SCI-induced respiratory dysfunctions and reactive neuroplasticity, the development of more clinically relevant contusion models is an important step to investigate posttraumatic lesional process and therapeutic strategies. In the case of a lateral C2 contusion, it has been reported that PhN inactivation remains incomplete and that functional recovery was very limited during the following weeks [14]. By contrast to C2 lateral hemisection, this ipsi PhN activity appears to arise rather from spared CPP pathways than from the reorganization of ipsi descending pathways. This is indeed indicated by the fact that stimulation of the ipsi ventrolateral column at C1 level did not elicit any response on the ipsi PhN and also by the observation that an additional contralateral C1 hemisection completely abolished the ipsi PhN activity [57]. A similar suppressive effect of a contralateral hemisection is also observed in the case of a contusion at the level of the PhMN nucleus [58] revealing thus a predominant role of CPP pathways. However, even in this case, transsynaptic tracing from the ipsi hemidiaphragm revealed an increased recruitment of interneurons rostral to the injury site one week after C3/C4 contusion [59]. Moreover, although the contribution of ipsi restorative pathways appears to be very limited in spontaneous conditions, it could be promoted by the adjunction of therapeutical strategies. Thus, in rats with a chronic lateral C2 contusion that received a transplantation of nasal olfactory ensheathing cells, stimulation of C1 ipsi ventrolateral column evoked an ipsi PhN response and moreover the recovered PhN activity was suppressed by an ipsi C1 hemisection, suggesting that PhMNs in these animals are reinnervated by ipsi descending pathways [57].

#### 2.2.4. Other Spinal Respiratory Plasticity Processes

It has been speculated that the SC contains neurons able to generate respiratory drive and rhythm like it is the case in the brainstem [60]. Thus, in cats, PhN output can be partially maintained after lesioning the medullary respiratory centers [61] or after a C1 transection [62]. However, such intrinsic spinal inspiratory activity independent of the medullary respiratory descending pathways has not been confirmed in medullospinal preparation of rats [63]. It has been suggested that spinal respiratory rhythm generator, although inefficient in intact rodents, could be induced by neuroplasticity processes following chronic SCI [60]. However, in adult rats 3 months after a lateral C2 SCI, we observed that the ipsi recovered PhN activity was fully abolished by a supplementary complete ipsi section at C1 level that interrupts all medullospinal ipsi drive [18]. It may rather exclude the possibility that SRI below C1 may themselves generate the recovered respiratory activity in absence of any supraspinal command. Thus, even if spared C1-C2 inspiratory neurons are recruited after chronic SCI, their activity may be dependent on the connections they receive from the collaterals of bulbospinal neurons.

In addition to injured pathways rerouting and to spared pathways reinforcement, other neuroplasticity processes may potentially contribute to the respiratory recovery, especially at the level of the ipsi phrenic nucleus. Thus, using an injection of a Sindbus viral vector expressing Channelrhodopsin in the phrenic nucleus of C2 injured animals, Alilain et al. 2008 have shown that light activation of ChR2-expressing phrenic cells was sufficient to entail durably recovery of diaphragmatic activity. This NMDA-dependent respiratory recovery may be attributed to a form of respiratory neuroplasticity through a synaptic reinforcement to phrenic cells (PhMN and/or SRI) [54]. In the phrenic nucleus of chronically hemisected rodents, previous studies have indeed shown an increase in glutamatergic terminal length and in NMDA 2A subunit [64, 65], in addition to the reorganization and reinforcement of the serotoninergic modulatory pathways as indicated by an increase of presynaptic 5-HT terminals and of its receptor 5HT2A [44, 66–68]. Such PhMN plasticity processes may have their origin in the preconditions established by chronic SCI, as an increase in BDNF/TrkB signaling. Experiments using alteration of the BDNF/TrkB signaling in hemisected rats (by BDNF or TrkB-Fc infusion in C4 using an implanted miniosmotic pump) indicate that BDNF/TrkB signaling in PhMN pool plays indeed a critical role in functional postinjury neuroplasticity processes [69]. Thus, these postlesion changes at the level of the phrenic cells and synapses may be efficient to reorganize respiratory descending pathways and to participate in the observed ventilatory recovery.

The importance of postlesion pathways reorganization following SCI raises the question whether they may only rely on preexisting neurons (damaged or spared) or if they may also putatively involve the production of new neurons as SRI or phrenic MN in the case of the respiratory system. Numerous studies have shown that the proliferation of neural progenitors in the neurogenic niches of the adult forebrain is stimulated after various types of brain injury, including stroke, seizure, or traumatic brain injury [70–74]. These newly generated neurons persist over time and might participate in the cognitive recovery [75]. The adult Mammalian SC is however a nonneurogenic tissue in physiological conditions. Although it retains multipotent neural stem cells that could generate functional neurons* in vitro* [76, 77], their potential is mainly restricted to the glial lineage* in vivo* [78, 79]. SCI is well known to induce the formation of reactive astrocytes and the infiltration of immune cells in the vicinity of the lesion site [80–83], but whether SCI also induces the production of new neurons* in vivo* has been controversial until recently. Studies have shown that spinal neurogenesis occurs to a limited extent after SCI [84–86] but that it could be stimulated by experimental intervention [87]. Overall, available data suggest that the extent of neurogenesis at the spinal level after SCI depends on the localization and severity of the injury [87, 88]. Recently, using the C2 unilateral injury model known to induce neuroplasticity, we have shown that neurogenesis remains absent in the injured SC [89]. Moreover, in these same injured animals, neurogenesis is notably decreased in the hippocampal dentate gyrus and this is correlated with brain inflammation suggesting that forebrain is sensitive to SCI. These results were confirmed by another group using a thoracic contusion model [90]. Thus, injury-induced neurogenesis does not appear to be an effective plasticity process participating in SC anatomical reorganization and to functional spontaneous recovery.

## 3. Part II: The Molecular Basis of Respiratory Neurons Plasticity following C2 SCI

Postinjury reactive plasticity processes and respiratory recovery rely in part on injured respiratory neurons and pathways. It supposes that the injured neurons may be able to both (1) overcome the neuron insult and survive and (2) reactivate neuronal growth programs to promote axon sprouting. This raises the question of the molecular processes that will sustain and coordinate such neuronal growth and survival. The molecular mechanisms of postinjury regenerative processes have mostly been studied in the PNS but are less well characterized in the CNS. They could involve an intrinsic postlesion cell body response (CBR) based on the upregulation of specific cell factors, as heat shock proteins (HSPs), that will assume a dual function in neuroprotection and neuroplasticity. They may also depend on the activation of specific signaling pathways depending on external cues, as neurotrophins, that are known to play a bipotential role in neuroprotective and neuroregenerative processes.

### 3.1. The Postlesion CBR and Its Role in Respiratory Neurons Plasticity

#### 3.1.1. Axotomy-Dependent Transcription Factors in Brainstem Neurons

**Figure 2:**
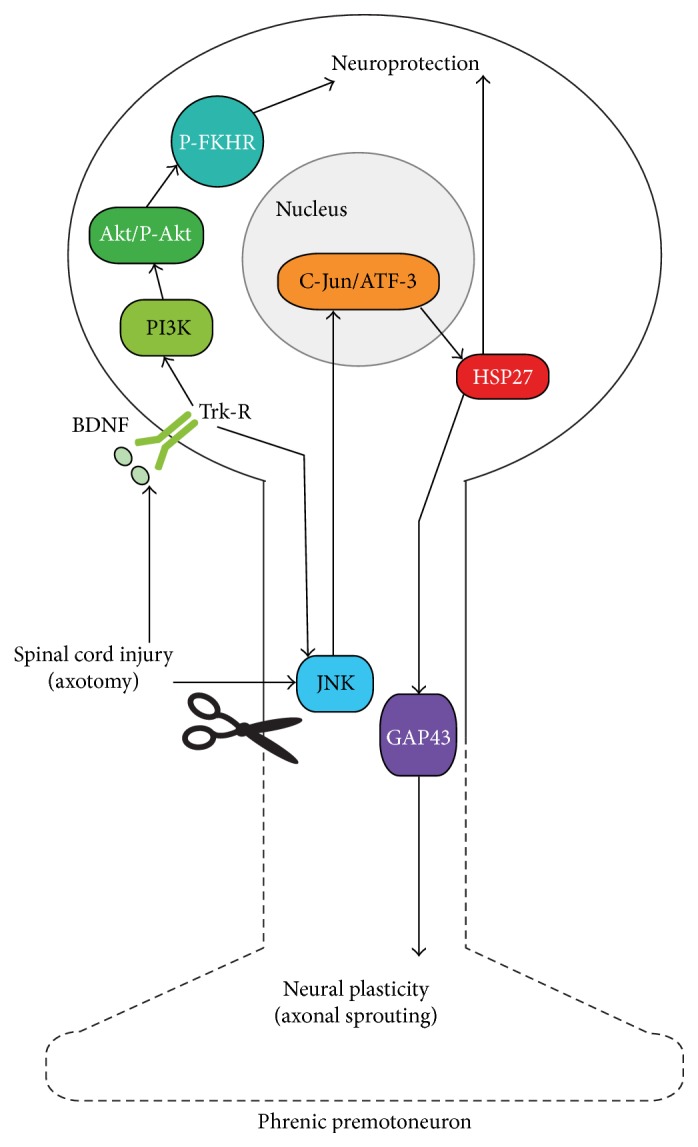
Diagram showing the induction of neuroprotection and neuroplasticity of phrenic premotoneurons after a cervical spinal cord injury. The injury-induced neuroplasticity through GAP43 expression involves JNK activation, c-Jun and ATF3 activation, and HSP27 induction. JNK can also be activated by tyrosine kinase receptor (Trk-R). The injury-induced neuroprotection involves BDNF increase, activation of TrK-R, activation of PI3K-Akt, and phosphorylation of FKHR. The pathway inducing HSP27 is also known to be involved in neuroprotection.

Survival and plasticity of injured neurons may be related, at least in part, to the intrinsic CBR to the axon damage and the induced cell stress. As shown in the PNS, axotomy triggers a rapid activation of JNK (C-Jun N-Terminal Kinase) at the injury site [91] and its retrograde transport from the injury site to the perikaryon where it induces both expression and phosphorylation of c-Jun, a component of the heterodimeric AP-1 transcription factor, followed by the induction of ATF-3 (activating transcription factor 3) [92] (Figure 2). These two transcription factors are considered as central regulators of the postlesion CBR that will orchestrate axonal outgrowth but also control the balance between cell death and survival. Thus, after nerve injury (that can entail nerve regeneration), the ability of injured neurons to regenerate their axon is correlated with a substantial upregulation of c-Jun and ATF-3 in injured neurons [91, 92]. For instance, after transection of the facial nerve, the suppression of c-Jun caused severe defects in several aspects of the axonal response and showed reduced target muscle reinnervation, indicating that c-Jun expression is essential for nerve fibers regeneration whereas its phosphorylation by JNKs appears less necessary [93, 94]. Similarly, following CNS neurons injury, a specific molecular CBR, including an upregulation of c-Jun and of some other axon growth effectors (as GAP-43), has been reported in some CNS neuronal populations [91]. This CBR may reveal an intrinsic growth potential which determines the neuron ability for axon regeneration if a growth permissive environment is provided, as, for instance, by a peripheral graft. Such molecular CBR may also promote axon sprouting in a standard environment. This is suggested by* in vitro* experiments showing that modulation of ATF3 and/or c-Jun in neuron cultures will alter the level of neurite sprouting and elongation [95, 96]. Thus, for a given CNS neuron population, the more or less efficiency to develop postlesion neuroplasticity and neuroregenerative processes may rely on their postlesion CBR intensity.

The level of the postlesion CBR will principally depend on the distance between the site of injury and the cell bodies, consistently with the better regenerative potential within a growth permissive environment observed for the neurons that are the nearest to the injury site (see Section 2). Thus, regarding corticospinal neurons, they are able to upregulate a range of growth-associated genes (and to regenerate within a peripheral nerve graft) following intracortical but not spinal cervical axotomy [97]. This differential CBR in link with the somatic distance to the injury and the regenerative potential is also shown by the fact that rubrospinal and some brainstem neurons (which are known to regenerate into cervical but not thoracic nerve transplants) respond to a cervical SCI by upregulating various regeneration-associated genes [28, 98, 99] but not to a thoracic SCI [100]. After thoracic lesioning, only thoracic propriospinal neurons (close to the SCI) are able to upregulate efficiently growth-associated genes whereas those located at the cervical level are not [101, 102]. However, in line with this higher CBR, thoracic propriospinal neurons are also more vulnerable and most of them will die after thoracic SCI, which is not the case for cervical ones [103]. Thus, the optimal soma/SCI distance for anatomical neuroplasticity will be a compromise between a sufficient proximity to favor growth-associated processes and a minimal distance to prevent cell death. In rodents with high cervical SCI, it may be the case for bulbospinal neurons as respiratory ones, which show both a high survival and an efficient regenerative response (see Section 2).

The SCI-soma distance is however not the sole factor accounting for the differential capability to upregulate growth-associated genes and to develop regenerative growth, as suggested by the more or less good regenerative potential within a nerve graft from various brainstem nuclei [32]. In order to investigate the postlesion CBR level of various populations of brainstem neurons, we first assessed the presence of c-Jun in axotomized respiratory neurons of the rVRG by comparison to other injured cell populations in the medulla [104]. We found that most axotomized respiratory neurons (around 60%) upregulate c-Jun after a C2 SCI (Figure 2) but it was also the case for the other damaged brainstem nuclei, suggesting that c-Jun induction may not be sufficient by itself to determine and trigger successful postlesion CBR and axon growth. Besides its important role for axonal growth, c-Jun is also a powerful mediator of axotomy-induced cell death, via JNK [93, 105]. For instance, suppression of c-Jun by siRNA enhances the survival of retinal ganglion cell following optic nerve cut [106] while overexpression of c-Jun enhances death of injured Purkinje neurons [107]. However, in the respiratory system, most axotomized bulbospinal neurons survive after cervical SCI despite their c-Jun expression indicating that they are prevented from c-Jun-induced cell death. The switch between neuronal death and regenerative growth will also depend on other cell cues which will allow us to imbalance the postinjury neuronal fate toward neuroprotective and neuroregenerative processes rather than neurodegeneration.

#### 3.1.2. HSP27: An Antiapoptotic and Growth-Promoting Factor

The downstream signaling induced by c-Jun and ATF3 leads to the induction of various effectors of postlesional neuronal response, among which are members of the HSPs family, notably HSP27. On the model of the superior nerve ganglion neurons, it has been shown that the ATF-3/c-Jun dependent transcription will produce the antiapoptotic factor HSP27 which in turn will inhibit JNK/c-Jun-induced apoptosis [108] (Figure 2). HSP27 expression is known to be elevated in patients with degenerative disorders or stroke [109, 110] and its induction and phosphorylation exert neuroprotection in various cerebral insults [111]. Thus, HSP27 intravenous injection has been shown to prevent cell death following cerebral ischemia [112, 113]. Following nerve injury, HSP27 induction promotes neuronal survival [114] and it also favors axon growth and regeneration in the PNS [115, 116]. Following thoracic contusion in rats, a microarray analysis of the damaged tissue (24 h after injury) found the highest gene induction (4.22x) for HSP27 [117]. In a similar SCI model, such specific upregulation of HSP27 (in addition to ATF-3) has also been reported at the cellular level by qRT-PCR (for 89 genes) performed after laser microdissection [102]. This latter study reported moreover that HSP27 expression was sustained only in thoracic interneurons but not in cervical ones, in relation to their proximity and their greater regenerative potential.

In order to evaluate the postlesion CBR in the rVRG by comparison to other brainstem nuclei, we have investigated the presence of HSP27 in axotomized respiratory neurons several days after a C2 SCI. We have reported that, by contrast to c-Jun, HSP27 was differentially induced between the different injured brainstem nuclei, principally in the rVRG, the dorsal Gigantocellularis, and vestibular nuclei but seldom in the raphe nuclei and ventral Gi [104], in link with a different regenerative potential reported between these different neuron populations. Moreover, also in contrast to c-Jun, HSP27 was expressed only in a few axotomized neurons within a given neural group, about 20% in the rVRG, pointing out a heterogeneous postlesion CBR between the respiratory neurons (Figure 2). Similarly, a previous report detected HSP27 in only 6% of the axotomized retinal ganglion cells [115]. Such heterogeneous CBR between CNS axotomized neurons has also been shown for NO Synthase (NOS) [118], another factor known to be associated with CBR and to play a role in axon growth [119]. However, in the case of the injured retinal cells, although HSP27 was detected only in 6% of the ganglion cells, this value reaches 30% for those regenerating in a peripheral graft [115]. Altogether, these data suggest that the presence of HSP27 in axotomized neurons may be a good indicator of their growing competence and of their putative contribution to postinjury anatomic plasticity processes. Besides HSP27, an important mediator of the postaxotomy CBR is Stat3 which is activated by axotomy via JNK and which has both neuroprotective and regenerative roles after axotomy [120, 121].

After CNS injury, the ability of injured axon to regrowth may depend not only on the level of the postlesion CBR but also on its duration that has to be long enough to permit an effective pathway rerouting and rewiring. Thus, some brainstem neurons lose their potential to regenerate in permissive conditions at longer postinjury intervals [32]. Thus, the evolution of the CBR during the weeks and months following the injury may influence the regenerative processes associated with anatomical plasticity and the efficiency of reparative strategies applied after a chronic SCI [98]. After chronic cervical SCI, a long-lasting expression of c-Jun and NOS has been shown in some vestibulospinal neurons [118], known for their good regenerative potential in chronic conditions [26, 32]. Therefore, in order to investigate whether axotomized respiratory neurons may also maintain durably their growth-reactive state after a chronic spinal C2 hemisection we have analyzed the evolution of some markers of the postlesion CBR in the rVRG and by comparison in other medullospinal regions. We found that HSP27 was still expressed at one month after SCI in approximately 20% of the axotomized respiratory neurons, similar to the one-week post-SCI time point, suggesting that this subset of HSP27 immunoreactive neurons conserve their responsive state for at least one month (manuscript in preparation). Interestingly, we observed that the same subset of HSP27+ respiratory neurons also express durably other growth-promoting genes (c-Jun and NOS) and moreover that they will downregulate the protein NeuN, a marker of neural mature state (manuscript in preparation), a fact previously reported for axotomized facial motoneurons [122]. Thus, one can identify a subset of medullary respiratory neurons that remain durably in a growth-reactive state after a chronic SCI, HSP27 appearing to be an early cell indicator of this prolonged CBR. One can postulate that this rVRG neuronal subpopulation exhibiting a long-lasting CBR may correspond to that potentially involved in neuroregenerative processes and in long-term respiratory pathways plasticity although the link between these growth-reactive states and axonal rewiring remains to be further established.

### 3.2. The BDNF/AKT/FKHR Pathway

The damaged neuron will integrate both internal and external cues to determine its cell fate after injury. Among the different molecular factors involved in neuroprotection and activated by external cues after injury, the serine-threonine kinase Akt plays a critical role in controlling the balance between survival and apoptosis in CNS [123–125] (Figure 2). After CNS injury, including SCI, several studies showed an increase in Akt phosphorylation suggesting its role in protecting the injured nervous tissue [126–136]. Akt, once phosphorylated by phosphatidylinositol 3-kinase (PI3K), promotes cell survival and prevents apoptosis by phosphorylating and inactivating several apoptosis-inducing factors, including forkhead transcription factors (FKHR) [123, 125]. Furthermore, the use of PI3K specific inhibitor compounds like LY294002 [137] suppresses brain neuroprotection after cerebral ischemia or SCI by inhibiting phosphorylation of FKHR [138, 139]. These treated animals developed a more severe trauma compared to untreated ones. While injury-induced activation of Akt appears to be a neuroprotective molecular event occurring at the injured spinal level, Akt has also been reported to be as key mediator of several aspects of neurite outgrowth and elongation, a process involving the physical interaction between Akt and HSP27 [140]. One can therefore also address the question of the Akt role at the level of the supraspinal structures where it may operate by promoting both neuroprotection and growth processes and thus contribute to the spontaneous postlesion recovery.

Using the cervical C2 unilateral SCI model, we initially screened for supraspinal molecular signaling and demonstrated thus that the PI3K-Akt signaling pathway was activated in the medulla whereas its proapoptotic downstream target, FKHR, was inactivated [38] (Figure 2). Retrograde labeling of medullary injured premotoneurons, including respiratory ones which project to PhMN, revealed that these injured neurons survived to the C2 SCI and that they were positive for the neuroprotective signaling factor [38]. To further explore the functional importance of this supraspinal cell survival signaling, we demonstrate that medullary inhibition of PI3K-Akt, by cerebral infusion of the LY294002 PI3K inhibitor, activates one of its death-promoting downstream targets, Fas ligand, and prevents the ipsi spontaneous respiratory recovery normally observed within three weeks [38]. These findings provide novel evidence for supraspinal cellular contribution to the spontaneous respiratory recovery after partial SCI. On the model of brain ischemic injury, inhibition of PI3K had however no effect on Hsp27-mediated neuroprotection, suggesting that AKT/FKHR and HSP27 are two distinct cell pathways to promote cell survival [141].

Upstream of PI3K-Akt, BDNF, a PI3K-Akt activator, is known to influence the plasticity of the respiratory network after SCI [69, 142] and has been found upregulated in the injured SC [143]. Treatment of the injured SC or cell bodies of injured axons with BDNF has been shown to be efficient in favoring survival and neuroplasticity [144–151]. Thus, at the supraspinal level, it has been shown that BDNF prevents atrophy of rubrospinal neurons in adult rats after cervical SCI and also stimulates and sustains the expression of regeneration-associated genes [152]. In the medulla, we found that BDNF levels significantly increased as a function of time postinjury [38]. It suggests that BDNF cell signaling may contribute to the durable growth-promoting and neuroprotective cell response necessary to the long-term postlesion plasticity processes. It is interesting to note that a common BDNF/PI3K-Akt cell survival signaling pathway exists at both spinal and supraspinal levels to facilitate spontaneous recovery of the respiratory function.

## 4. Conclusion

Respiratory recovery following high cervical SCI has generally been attributed to the reinforcement of spared respiratory pathways, as for the classical “crossed phrenic pathways,” so that the contribution of the damaged neurons has so far been underestimated. The SCI may however promote an intrinsic cell body reaction by at least a subpopulation of the axotomized neurons in addition to a response to various external cues such as BDNF, in order to induce a neuronal shift toward an antiapoptotic and growth-promoting reactive state. Thus, a subset of injured respiratory neurons may spontaneously develop axon collateral sprouting at the level of the C1 grey matter and may contact propriospinal neurons to form new restorative pathways. In the case of a high cervical SCI, these neuroplastic changes may be fostered by the fact that respiratory damaged neurons reside in proximity of the injury and of their main motoneuron target. Altogether, respiratory pathways regrowth and reorganization may contribute to the spontaneous recovery observed after a chronic unilateral SCI in animal models. It may also explain why restorative strategies applied to hemisected rats can markedly improve the function of the ipsi hemidiaphragm. Thus, transplantation of olfactory ensheathing cells applied at the SC level [43, 153] entails a partial restoration of respiratory function [57, 154]. Similarly, partial recovery of respiratory function has been reported in hemisected rodent in which a peripheral nerve graft was applied to bridge the injury and allow the regeneration of respiratory medullary pathways toward their PhMN targets [37]. It points out the fact that the survival and growth-competence of the damaged respiratory and other bulbospinal pathways may be further exploited to develop repair strategies designed to humans.

## Abbreviations

SC: Spinal cord
SCI: Spinal cord injury
PhMN: Phrenic motoneurons
PhN: Phrenic nerve
rVRG: Rostral ventral respiratory group
SRI: Spinal respiratory interneurons
CNS: Central nervous system
PNS: Peripheral nervous system
CPP: Crossed phrenic pathways
CBR: Cell body response
Ipsi: Ipsilateral.

## References

[1] Ramer L. M., Ramer M. S., Bradbury E. J. (2014). Restoring function after spinal cord injury: towards clinical translation of experimental strategies. *The Lancet Neurology*.

[2] Bareyre F. M., Kerschensteiner M., Raineteau O., Mettenleiter T. C., Weinmann O., Schwab M. E. (2004). The injured spinal cord spontaneously forms a new intraspinal circuit in adult rats. *Nature Neuroscience*.

[3] Hoh D. J., Mercier L. M., Hussey S. P., Lane M. A. (2013). Respiration following spinal cord injury: evidence for human neuroplasticity. *Respiratory Physiology and Neurobiology*.

[4] Kastner A., Gauthier P. (2008). Are rodents an appropriate pre-clinical model for treating spinal cord injury? Examples from the respiratory system. *Experimental Neurology*.

[5] Warren P. M., Alilain W. J. (2014). The challenges of respiratory motor system recovery following cervical spinal cord injury. *Progress in Brain Research*.

[6] Lane M. A. (2011). Spinal respiratory motoneurons and interneurons. *Respiratory Physiology and Neurobiology*.

[7] Hilaire G., Pásaro R. (2003). Genesis and control of the respiratory rhythm in adult mammals. *News in Physiological Sciences*.

[8] Vinit S., Kastner A. (2009). Descending bulbospinal pathways and recovery of respiratory motor function following spinal cord injury. *Respiratory Physiology and Neurobiology*.

[9] Darlot F., Cayetanot F., Gauthier P., Matarazzo V., Kastner A. (2012). Extensive respiratory plasticity after cervical spinal cord injury in rats: axonal sprouting and rerouting of ventrolateral bulbospinal pathways. *Experimental Neurology*.

[10] Lipski J., Zhang X., Kruszewska B., Kanjhan R. (1994). Morphological study of long axonal projections of ventral medullary inspiratory neurons in the rat. *Brain Research*.

[11] Boulenguez P., Gauthier P., Kastner A. (2007). Respiratory neuron subpopulations and pathways potentially involved in the reactivation of phrenic motoneurons after C2 hemisection. *Brain Research*.

[12] Goshgarian H. G. (2003). Invited review: the crossed phrenic phenomenon: a model for plasticity in the respiratory pathways following spinal cord injury. *Journal of Applied Physiology*.

[13] Lane M. A., Lee K.-Z., Fuller D. D., Reier P. J. (2009). Spinal circuitry and respiratory recovery following spinal cord injury. *Respiratory Physiology and Neurobiology*.

[14] Baussart B., Stamegna J. C., Polentes J., Tadié M., Gauthier P. (2006). A new model of upper cervical spinal contusion inducing a persistent unilateral diaphragmatic deficit in the adult rat. *Neurobiology of Disease*.

[15] Vinit S., Stamegna J.-C., Boulenguez P., Gauthier P., Kastner A. (2007). Restorative respiratory pathways after partial cervical spinal cord injury: role of ipsilateral phrenic afferents. *European Journal of Neuroscience*.

[16] Fuller D. D., Golder F. J., Olson E. B., Mitchell G. S. (2006). Recovery of phrenic activity and ventilation after cervical spinal hemisection in rats. *Journal of Applied Physiology*.

[17] Nantwi K. D., El-Bohy A. A., Schrimsher G. W., Reier P. J., Goshgarian H. G. (1999). Spontaneous functional recovery in a paralyzed hemidiaphragm following upper cervical spinal cord injury in adult rats. *Neurorehabilitation and Neural Repair*.

[18] Vinit S., Darlot F., Stamegna J.-C., Sanchez P., Gauthier P., Kastner A. (2008). Long-term reorganization of respiratory pathways after partial cervical spinal cord injury. *European Journal of Neuroscience*.

[19] Fouad K., Pedersen V., Schwab M. E., Brösamle C. (2001). Cervical sprouting of corticospinal fibers after thoracic spinal cord injury accompanies shifts in evoked motor responses. *Current Biology*.

[20] Steward O., Zheng B., Tessier-Lavigne M., Hofstadter M., Sharp K., Yee K. M. (2008). Regenerative growth of corticospinal tract axons via the ventral column after spinal cord injury in mice. *Journal of Neuroscience*.

[21] Girgis J., Merrett D., Kirkland S., Metz G. A. S., Verge V., Fouad K. (2007). Reaching training in rats with spinal cord injury promotes plasticity and task specific recovery. *Brain*.

[22] Weishaupt N., Li S., Di Pardo A., Sipione S., Fouad K. (2013). Synergistic effects of BDNF and rehabilitative training on recovery after cervical spinal cord injury. *Behavioural Brain Research*.

[23] Blesch A., Yang H., Weidner N., Hoang A., Otero D. (2004). Axonal responses to cellularly delivered NT-4/5 after spinal cord injury. *Molecular and Cellular Neuroscience*.

[24] Kim J.-E., Liu B. P., Park J. H., Strittmatter S. M. (2004). Nogo-66 receptor prevents raphespinal and rubrospinal axon regeneration and limits functional recovery from spinal cord injury. *Neuron*.

[25] López-Vales R., Forés J., Navarro X., Verdú E. (2007). Chronic transplantation of olfactory ensheathing cells promotes partial recovery after complete spinal cord transection in the rat. *GLIA*.

[26] Vavrek R., Pearse D. D., Fouad K. (2007). Neuronal populations capable of regeneration following a combined treatment in rats with spinal cord transection. *Journal of Neurotrauma*.

[27] Wakabayashi Y., Komori H., Kawa-Uchi T. (2001). Functional recovery and regeneration of descending tracts in rats after spinal cord transection in infancy. *Spine*.

[28] Jenkins R., Tetzlaff W., Hunt S. P. (1993). Differential expression of immediate early genes in rubrospinal neurons following axotomy in rat. *European Journal of Neuroscience*.

[29] Blits B., Dijkhuizen P. A., Boer G. J., Verhaagen J. (2000). Intercostal nerve implants transduced with an adenoviral vector encoding neurotrophin-3 promote regrowth of injured rat corticospinal tract fibers and improve hindlimb function. *Experimental Neurology*.

[30] Decherchi P., Gauthier P. (2002). Regeneration of acutely and chronically injured descending respiratory pathways within post-traumatic nerve grafts. *Neuroscience*.

[31] Xu X. M., Zhang S.-X., Li H., Aebischer P., Bunge M. B. (1999). Regrowth of axons into the distal spinal cord through a Schwann-cell-seeded mini-channel implanted into hemisected adult rat spinal cord. *European Journal of Neuroscience*.

[32] Ye J.-H., Houle J. D. (1997). Treatment of the chronically injured spinal cord with neurotrophic factors can promote axonal regeneration from supraspinal neurons. *Experimental Neurology*.

[33] Richardson P. M., Issa V. M. K., Aguayo A. J. (1984). Regeneration of long spinal axons in the rat. *Journal of Neurocytology*.

[34] Filli L., Engmann A. K., Zörner B. (2014). Bridging the gap: a reticulo-propriospinal detour bypassing an incomplete spinal cord injury. *Journal of Neuroscience*.

[35] Ballermann M., Fouad K. (2006). Spontaneous locomotor recovery in spinal cord injured rats is accompanied by anatomical plasticity of reticulospinal fibers. *European Journal of Neuroscience*.

[36] Decherchi P., Gauthier P. (1996). In vitro pre-degenerated nerve autografts support CNS axonal regeneration. *Brain Research*.

[37] Alilain W. J., Horn K. P., Hu H., Dick T. E., Silver J. (2011). Functional regeneration of respiratory pathways after spinal cord injury. *Nature*.

[38] Felix M. S., Bauer S., Darlot F. (2014). Activation of Akt/FKHR in the medulla oblongata contributes to spontaneous respiratory recovery after incomplete spinal cord injury in adult rats. *Neurobiology of Disease*.

[39] Goshgarian H. G., Ellenberger H. H., Feldman J. L. (1991). Decussation of bulbospinal respiratory axons at the level of the phrenic nuclei in adult rats: a possible substrate for the crossed phrenic phenomenon. *Experimental Neurology*.

[40] Lipski J., Duffin J., Kruszewska B., Zhang X. (1993). Upper cervical inspiratory neurons in the rat: an electrophysiological and morphological study. *Experimental Brain Research*.

[41] Fuller D. D., Doperalski N. J., Dougherty B. J., Sandhu M. S., Bolser D. C., Reier P. J. (2008). Modest spontaneous recovery of ventilation following chronic high cervical hemisection in rats. *Experimental Neurology*.

[42] Ghali M. G. Z., Marchenko V. (2015). Dynamic changes in phrenic motor output following high cervical hemisection in the decerebrate rat. *Experimental Neurology*.

[43] Li Y., Decherchi P., Raisman G. (2003). Transplantation of olfactory ensheathing cells into spinal cord lesions restores breathing and climbing. *Journal of Neuroscience*.

[44] Golder F. J., Mitchell G. S. (2005). Spinal synaptic enhancement with acute intermittent hypoxia improves respiratory function after chronic cervical spinal cord injury. *The Journal of Neuroscience*.

[45] Lu F., Qin C., Foreman R. D., Farber J. P. (2004). Chemical activation of C1-C2 spinal neurons modulates intercostal and phrenic nerve activity in rats. *American Journal of Physiology—Regulatory Integrative and Comparative Physiology*.

[46] Oku Y., Okabe A., Hayakawa T., Okada Y. (2008). Respiratory neuron group in the high cervical spinal cord discovered by optical imaging. *NeuroReport*.

[47] Tian G.-F., Duffin J. (1996). Connections from upper cervical inspiratory neurons to phrenic and intercostal motoneurons studied with cross-correlation in the decerebrate rat. *Experimental Brain Research*.

[48] Tian G.-F., Duffin J. (1996). Spinal connections of ventral-group bulbospinal inspiratory neurons studied with cross-correlation in the decerebrate rat. *Experimental Brain Research*.

[49] Dobbins E. G., Feldman J. L. (1994). Brainstem network controlling descending drive to phrenic motoneurons in rat. *Journal of Comparative Neurology*.

[50] Lane M. A., Fuller D. D., White T. E., Reier P. J. (2008). Respiratory neuroplasticity and cervical spinal cord injury: translational perspectives. *Trends in Neurosciences*.

[51] Qiu K., Lane M. A., Lee K. Z., Reier P. J., Fuller D. D. (2010). The phrenic motor nucleus in the adult mouse. *Experimental Neurology*.

[52] Fuller D. D., Sandhu M. S., Doperalski N. J. (2009). Graded unilateral cervical spinal cord injury and respiratory motor recovery. *Respiratory Physiology and Neurobiology*.

[53] Courtine G., Song B., Roy R. R. (2008). Recovery of supraspinal control of stepping via indirect propriospinal relay connections after spinal cord injury. *Nature Medicine*.

[54] Alilain W. J., Li X., Horn K. P. (2008). Light-induced rescue of breathing after spinal cord injury. *Journal of Neuroscience*.

[55] Lee K.-Z., Dougherty B. J., Sandhu M. S., Lane M. A., Reier P. J., Fuller D. D. (2013). Phrenic motoneuron discharge patterns following chronic cervical spinal cord injury. *Experimental Neurology*.

[56] Sandhu M. S., Dougherty B. J., Lane M. A. (2009). Respiratory recovery following high cervical hemisection. *Respiratory Physiology and Neurobiology*.

[57] Stamegna J. C., Felix M. S., Roux-Peyronnet J. (2011). Nasal OEC transplantation promotes respiratory recovery in a subchronic rat model of cervical spinal cord contusion. *Experimental Neurology*.

[58] Awad B. I., Warren P. M., Steinmetz M. P., Alilain W. J. (2013). The role of the crossed phrenic pathway after cervical contusion injury and a new model to evaluate therapeutic interventions. *Experimental Neurology*.

[59] Lane M. A., Lee K.-Z., Salazar K. (2012). Respiratory function following bilateral mid-cervical contusion injury in the adult rat. *Experimental Neurology*.

[60] Warren P. M., Awad B. I., Alilain W. J. (2014). Drawing breath without the command of effectors: the control of respiration following spinal cord injury. *Respiratory Physiology and Neurobiology*.

[61] Speck D. F., Feldman J. L. (1982). The effects of microstimulation and microlesions in the ventral and dorsal respiratory groups in medulla of cat. *The Journal of Neuroscience*.

[62] Aoki M., Mori S., Kawahara K., Watanabe H., Ebata N. (1980). Generation of spontaneous respiratory rhythm in high spinal cats. *Brain Research*.

[63] Jones S. E., Saad M., Lewis D. I., Subramanian H. H., Dutschmann M. (2012). The nucleus retroambiguus as possible site for inspiratory rhythm generation caudal to obex. *Respiratory Physiology and Neurobiology*.

[64] Alilain W. J., Goshgarian H. G. (2008). Glutamate receptor plasticity and activity-regulated cytoskeletal associated protein regulation in the phrenic motor nucleus may mediate spontaneous recovery of the hemidiaphragm following chronic cervical spinal cord injury. *Experimental Neurology*.

[65] Tai Q., Goshgarian H. G. (1996). Ultrastructural quantitative analysis of glutamatergic and GABAergic synaptic terminals in the phrenic nucleus after spinal cord injury. *Journal of Comparative Neurology*.

[66] Fuller D. D., Baker-Herman T. L., Golder F. J., Doperalski N. J., Watters J. J., Mitchell G. S. (2005). Cervical spinal cord injury upregulates ventral spinal 5-HT2A receptors. *Journal of Neurotrauma*.

[67] Mantilla C. B., Bailey J. P., Zhan W.-Z., Sieck G. C. (2012). Phrenic motoneuron expression of serotonergic and glutamatergic receptors following upper cervical spinal cord injury. *Experimental Neurology*.

[68] Tai Q., Palazzolo K. L., Goshgarian H. G. (1997). Synaptic plasticity of 5-hydroxytryptamine-immunoreactive terminals in the phrenic nucleus following spinal cord injury: a quantitative electron microscopic analysis. *Journal of Comparative Neurology*.

[69] Mantilla C. B., Gransee H. M., Zhan W.-Z., Sieck G. C. (2013). Motoneuron BDNF/TrkB signaling enhances functional recovery after cervical spinal cord injury. *Experimental Neurology*.

[70] Kernie S. G., Erwin T. M., Parada L. F. (2001). Brain remodeling due to neuronal and astrocytic proliferation after controlled cortical injury in mice. *Journal of Neuroscience Research*.

[71] Rola R., Mizumatsu S., Otsuka S. (2006). Alterations in hippocampal neurogenesis following traumatic brain injury in mice. *Experimental Neurology*.

[72] Urrea C., Castellanos D. A., Sagen J., Tsoulfas P., Bramlett H. M., Dietrich W. D. (2007). Widespread cellular proliferation and focal neurogenesis after traumatic brain injury in the rat. *Restorative Neurology and Neuroscience*.

[73] Sun D., McGinn M. J., Zhou Z., Harvey H. B., Bullock M. R., Colello R. J. (2007). Anatomical integration of newly generated dentate granule neurons following traumatic brain injury in adult rats and its association to cognitive recovery. *Experimental Neurology*.

[74] Yu T.-S., Zhang G., Liebl D. J., Kernie S. G. (2008). Traumatic brain injury-induced hippocampal neurogenesis requires activation of early nestin-expressing progenitors. *Journal of Neuroscience*.

[75] Blaiss C. A., Yu T.-S., Zhang G. (2011). Temporally specified genetic ablation of neurogenesis impairs cognitive recovery after traumatic brain injury. *The Journal of Neuroscience*.

[76] Dromard C., Guillon H., Rigau V. (2008). Adult human spinal cord harbors neural precursor cells that generate neurons and glial cells in vitro. *Journal of Neuroscience Research*.

[77] Meletis K., Barnabé-Heider F., Carlén M. (2008). Spinal cord injury reveals multilineage differentiation of ependymal cells. *PLoS Biology*.

[78] Horner P. J., Power A. E., Kempermann G. (2000). Proliferation and differentiation of progenitor cells throughout the intact adult rat spinal cord. *Journal of Neuroscience*.

[79] Wrathall J. R., Lytle J. M. (2008). Stem cells in spinal cord injury. *Disease Markers*.

[80] Beck K. D., Nguyen H. X., Galvan M. D., Salazar D. L., Woodruff T. M., Anderson A. J. (2010). Quantitative analysis of cellular inflammation after traumatic spinal cord injury: evidence for a multiphasic inflammatory response in the acute to chronic environment. *Brain*.

[81] Rolls A., Shechter R., Schwartz M. (2009). The bright side of the glial scar in CNS repair. *Nature Reviews Neuroscience*.

[82] Yang H., Lu P., McKay H. M. (2006). Endogenous neurogenesis replaces oligodendrocytes and astrocytes after primate spinal cord injury. *The Journal of Neuroscience*.

[83] Horky L. L., Galimi F., Gage F. H., Horner P. J. (2006). Fate of endogenous stem/progenitor cells following spinal cord injury. *Journal of Comparative Neurology*.

[84] Chi L., Ke Y., Luo C. (2006). Motor neuron degeneration promotes neural progenitor cell proliferation, migration, and neurogenesis in the spinal cords of amyotrophic lateral sclerosis mice. *Stem Cells*.

[85] Ke Y., Chi L., Xu R., Luo C., Gozal D., Liu R. (2006). Early response of endogenous adult neural progenitor cells to acute spinal cord injury in mice. *STEM CELLS*.

[86] Shechter R., Ziv Y., Schwartz M. (2007). New GABAergic interneurons supported by myelin-specific T cells are formed in intact adult spinal cord. *Stem Cells*.

[87] Ohori Y., Yamamoto S.-I., Nagao M. (2006). Growth factor treatment and genetic manipulation stimulate neurogenesis and oligodendrogenesis by endogenous neural progenitors in the injured adult spinal cord. *Journal of Neuroscience*.

[88] Vessal M., Aycock A., Garton M. T., Ciferri M., Darian-Smith C. (2007). Adult neurogenesis in primate and rodent spinal cord: comparing a cervical dorsal rhizotomy with a dorsal column transection. *European Journal of Neuroscience*.

[89] Felix M.-S., Popa N., Djelloul M. (2012). Alteration of forebrain neurogenesis after cervical spinal cord injury in the adult rat. *Frontiers in Neuroscience*.

[90] Wu J., Zhao Z., Sabirzhanov B. (2014). Spinal cord injury causes brain inflammation associated with cognitive and affective changes: role of cell cycle pathways. *Journal of Neuroscience*.

[91] Herdegen T., Skene P., Bähr M. (1997). The c-Jun transcription factor—bipotential mediator of neuronal death, survival and regeneration. *Trends in Neurosciences*.

[92] Patodia S., Raivich G. (2012). Role of transcription factors in peripheral nerve regeneration. *Frontiers in Molecular Neuroscience*.

[93] Raivich G., Bohatschek M., Da Costa C. (2004). The AP-1 transcription factor c-Jun is required for efficient axonal regeneration. *Neuron*.

[94] Ruff C. A., Staak N., Patodia S. (2012). Neuronal c-Jun is required for successful axonal regeneration, but the effects of phosphorylation of its N-terminus are moderate. *Journal of Neurochemistry*.

[95] Pearson A. G., Gray C. W., Pearson J. F., Greenwood J. M., During M. J., Dragunow M. (2003). ATF3 enhances c-Jun-mediated neurite sprouting. *Molecular Brain Research*.

[96] Seijffers R., Allchorne A. J., Woolf C. J. (2006). The transcription factor ATF-3 promotes neurite outgrowth. *Molecular and Cellular Neuroscience*.

[97] Mason M. R. J., Lieberman A. R., Anderson P. N. (2003). Corticospinal neurons up-regulate a range of growth-associated genes following intracortical, but not spinal, axotomy. *European Journal of Neuroscience*.

[98] Houle J. D., Schramm P., Herdegen T. (1998). Trophic factor modulation of c-Jun expression in supraspinal neurons after chronic spinal cord injury. *Experimental Neurology*.

[99] Vinit S., Boulenguez P., Efthimiadi L., Stamegna J.-C., Gauthier P., Kastner A. (2005). Axotomized bulbospinal neurons express c-Jun after cervical spinal cord injury. *NeuroReport*.

[100] Fernandes K. J. L., Fan D.-P., Tsui B. J., Cassar S. L., Tetzlaff W. (1999). Influence of the axotomy to cell body distance in rat rubrospinal and spinal motoneurons: differential regulation of GAP-43, tubulins, and neurofilament-M. *Journal of Comparative Neurology*.

[101] Siebert J. R., Middelton F. A., Stelzner D. J. (2010). Intrinsic response of thoracic propriospinal neurons to axotomy. *BMC Neuroscience*.

[102] Siebert J. R., Middleton F. A., Stelzner D. J. (2010). Long descending cervical propriospinal neurons differ from thoracic propriospinal neurons in response to low thoracic spinal injury. *BMC Neuroscience*.

[103] Conta Steencken A. C., Smirnov I., Stelzner D. J. (2011). Cell survival or cell death: differential vulnerability of long descending and thoracic propriospinal neurons to low thoracic axotomy in the adult rat. *Neuroscience*.

[104] Vinit S., Darlot F., Aoulaïche H., Boulenguez P., Kastner A. (2011). Distinct expression of c-Jun and HSP27 in axotomized and spared bulbospinal neurons after cervical spinal cord injury. *Journal of Molecular Neuroscience*.

[105] Waetzig V., Zhao Y., Herdegen T. (2006). The bright side of JNKs-Multitalented mediators in neuronal sprouting, brain development and nerve fiber regeneration. *Progress in Neurobiology*.

[106] Lingor P., Koeberle P., Kügler S., Bähr M. (2005). Down-regulation of apoptosis mediators by RNAi inhibits axotomy-induced retinal ganglion cell death *in vivo*. *Brain*.

[107] Carulli D., Buffo A., Botta C., Altruda F., Strata P. (2002). Regenerative and survival capabilities of Purkinje cells overexpressing c-Jun. *European Journal of Neuroscience*.

[108] Nakagomi S., Suzuki Y., Namikawa K., Kiryu-Seo S., Kiyama H. (2003). Expression of the activating transcription factor 3 prevents c-Jun N-terminal kinase-induced neuronal death by promoting heat shock protein 27 expression and akt activation. *Journal of Neuroscience*.

[109] Costigan M., Mannion R. J., Kendall G. (1998). Heat shock protein 27: developmental regulation and expression after peripheral nerve injury. *The Journal of Neuroscience*.

[110] Read D. E., Gorman A. M. (2009). Heat shock protein 27 in neuronal survival and neurite outgrowth. *Biochemical and Biophysical Research Communications*.

[111] Stetler R. A., Gao Y., Signore A. P., Cao G., Chen J. (2009). HSP27: mechanisms of cellular protection against neuronal injury. *Current Molecular Medicine*.

[112] Shimada Y., Tanaka R., Shimura H., Yamashiro K., Urabe T., Hattori N. (2014). Phosphorylation enhances recombinant HSP27 neuroprotection against focal cerebral ischemia in mice. *Neuroscience*.

[113] Teramoto S., Shimura H., Tanaka R. (2013). Human-derived physiological heat shock protein 27 complex protects brain after focal cerebral ischemia in mice. *PLoS ONE*.

[114] Benn S. C., Perrelet D., Kato A. C. (2002). Hsp27 upregulation and phosphorylation is required for injured sensory and motor neuron survival. *Neuron*.

[115] Hebb M. O., Myers T. L., Clarke D. B. (2006). Enhanced expression of heat shock protein 27 is correlated with axonal regeneration in mature retinal ganglion cells. *Brain Research*.

[116] Williams K. L., Rahimtula M., Mearow K. M. (2006). Heat shock protein 27 is involved in neurite extension and branching of dorsal root ganglion neurons in vitro. *Journal of Neuroscience Research*.

[117] Tachibana T., Noguchi K., Ruda M. A. (2002). Analysis of gene expression following spinal cord injury in rat using complementary DNA microarray. *Neuroscience Letters*.

[118] Jin Y., Tay D., So K.-F., Wu W. (2000). Expression of c-jun in the lateral vestibular nucleus following spinal cord injury and peripheral nerve graft transplantation in adult rats. *Journal of Neurocytology*.

[119] Klöcker N., Jung M., Stuermer C. A. O., Bähr M. (2001). BDNF increases the number of axotomized rat retinal ganglion cells expressing GAP-43, L1, and TAG-1 mRNA—a supportive role for nitric oxide?. *Neurobiology of Disease*.

[120] Schweizer U., Gunnersen J., Karch C. (2002). Conditional gene ablation of Stat3 reveals differential signaling requirements for survival of motoneurons during development and after nerve injury in the adult. *The Journal of Cell Biology*.

[121] Sheu J. Y., Kulhanek D. J., Eckenstein F. P. (2000). Differential patterns of ERK and STAT3 phosphorylation after sciatic nerve transection in the rat. *Experimental Neurology*.

[122] McPhail L. T., McBride C. B., McGraw J., Steeves J. D., Tetzlaff W. (2004). Axotomy abolishes NeuN expression in facial but not rubrospinal neurons. *Experimental Neurology*.

[123] Burgering B. M., Kops G. J. (2002). Cell cycle and death control: long live Forkheads. *Trends in Biochemical Sciences*.

[124] Dudek H., Datta S. R., Franke T. F. (1997). Regulation of neuronal survival by the serine-threonine protein kinase Akt. *Science*.

[125] Brunet A., Bonni A., Zigmond M. J. (1999). Akt promotes cell survival by phosphorylating and inhibiting a Forkhead transcription factor. *Cell*.

[126] Ouyang Y.-B., Tan Y., Comb M. (1999). Survival- and death-promoting events after transient cerebral ischemia: phosphorylation of Akt, release of cytochrome C and Activation of caspase-like proteases. *Journal of Cerebral Blood Flow & Metabolism*.

[127] Namura S., Nagata I., Kikuchi H., Andreucci M., Alessandrini A. (2000). Serine-threonine protein kinase Akt does not mediate ischemic tolerance after global ischemia in the gerbil. *Journal of Cerebral Blood Flow and Metabolism*.

[128] Yano S., Morioka M., Fukunaga K. (2001). Activation of Akt/protein kinase B contributes to induction of ischemic tolerance in the CA1 subfield of gerbil hippocampus. *Journal of Cerebral Blood Flow and Metabolism*.

[129] Noshita N., Lewén A., Sugawara T., Chan P. H. (2002). Akt phosphorylation and neuronal survival after traumatic brain injury in mice. *Neurobiology of Disease*.

[130] Noshita N., Lewén A., Sugawara T., Chan P. H. (2001). Evidence of phosphorylation of Akt and neuronal survival after transient focal cerebral ischemia in mice. *Journal of Cerebral Blood Flow and Metabolism*.

[131] Yoshimoto T., Uchino H., He Q. P., Li P. A., Siesjö B. K. (2001). Cyclosporin A, but not FK506, prevents the downregulation of phosphorylated Akt after transient focal ischemia in the rat. *Brain Research*.

[132] Janelidze S., Hu B.-R., Siesjö P., Siesjö B. K. (2001). Alterations of Akt1 (PKB*α*) and p70^S6K^ in transient focal ischemia. *Neurobiology of Disease*.

[133] Yune T. Y., Park H. G., Lee J. Y., Oh T. H. (2008). Estrogen-induced Bcl-2 expression after spinal cord injury is mediated through phosphoinositide-3-kinase/Akt-dependent CREB activation. *Journal of Neurotrauma*.

[134] Shi T.-J. S., Huang P., Mulder J., Ceccatelli S., Hökfelt T. (2009). Expression of p-Akt in sensory neurons and spinal cord after peripheral nerve injury. *NeuroSignals*.

[135] Yu F., Sugawara T., Maier C. M., Hsieh L. B., Chan P. H. (2005). Akt/Bad signaling and motor neuron survival after spinal cord injury. *Neurobiology of Disease*.

[136] Zhao H., Shimohata T., Wang J. Q. (2005). Akt contributes to neuroprotection by hypothermia against cerebral ischemia in rats. *The Journal of Neuroscience*.

[137] Vlahos C. J., Matter W. F., Hui K. Y., Brown R. F. (1994). A specific inhibitor of phosphatidylinositol 3-kinase, 2-(4-morpholinyl)-8-phenyl-4H-1-benzopyran-4-one (LY294002). *The Journal of Biological Chemistry*.

[138] Zhan L., Li D., Liang D. (2012). Activation of Akt/FoxO and inactivation of MEK/ERK pathways contribute to induction of neuroprotection against transient global cerebral ischemia by delayed hypoxic postconditioning in adult rats. *Neuropharmacology*.

[139] Paterniti I., Esposito E., Mazzon E., Bramanti P., Cuzzocrea S. (2011). Evidence for the role of PI_3_-kinase-AKT-eNOS signalling pathway in secondary inflammatory process after spinal cord compression injury in mice. *European Journal of Neuroscience*.

[140] Read D. E., Gorman A. M. (2009). Involvement of Akt in neurite outgrowth. *Cellular and Molecular Life Sciences*.

[141] Stetler R. A., Cao G., Gao Y. (2008). Hsp27 protects against ischemic brain injury via attenuation of a novel stress-response cascade upstream of mitochondrial cell death signaling. *The Journal of Neuroscience*.

[142] Sieck G. C., Mantilla C. B. (2009). Role of neurotrophins in recovery of phrenic motor function following spinal cord injury. *Respiratory Physiology and Neurobiology*.

[143] Li X.-L., Zhang W., Zhou X. (2007). Temporal changes in the expression of some neurotrophins in spinal cord transected adult rats. *Neuropeptides*.

[144] Bregman B. S., McAtee M., Dai H. N., Kuhn P. L. (1997). Neurotrophic factors increase axonal growth after spinal cord injury and transplantation in the adult rat. *Experimental Neurology*.

[145] Coumans J. V., Lin T. T.-S., Hai Ning Dai (2001). Axonal regeneration and functional recovery after complete spinal cord transection in rats by delayed treatment with transplants and neurotrophins. *The Journal of Neuroscience*.

[146] Kobayashi N. R., Fan D.-P., Giehl K. M., Bedard A. M., Wiegand S. J., Tetzlaff W. (1997). BDNF and NT-4/5 prevent atrophy of rat rubrospinal neurons after cervical axotomy, stimulate GAP-43 and Talpha1-tubulin mRNA expression, and promote axonal regeneration. *The Journal of Neuroscience*.

[147] Hammond E. N. L., Tetzlaff W., Mestres P., Giehl K. M. (1999). BDNF, but not NT-3, promotes long-term survival of axotomized adult rat corticospinal neurons in vivo. *NeuroReport*.

[148] Schnell L., Schneider R., Kolbeck R., Barde Y.-A., Schwab M. E. (1994). Neurotrophin-3 enhances sprouting of corticospinal tract during development and after adult spinal cord lesion. *Nature*.

[149] Plunet W., Kwon B. K., Tetzlaff W. (2002). Promoting axonal regeneration in the central nervous system by enhancing the cell body response to axotomy. *Journal of Neuroscience Research*.

[150] Vavrek R., Girgis J., Tetzlaff W., Hiebert G. W., Fouad K. (2006). BDNF promotes connections of corticospinal neurons onto spared descending interneurons in spinal cord injured rats. *Brain*.

[151] Giehl K. M., Tetzlaff W. (1996). BDNF and NT-3, but not NGF, prevent axotomy-induced death of rat corticospinal neurons in vivo. *European Journal of Neuroscience*.

[152] Tetzlaff W., Kobayashi N. R., Giehl K. M. G., Tsui B. J., Cassar S. L., Bedard A. M. (1994). Response of rubrospinal and corticospinal neurons to injury and neurotrophins. *Progress in Brain Research*.

[153] Polentes J., Stamegna J. C., Nieto-Sampedro M., Gauthier P. (2004). Phrenic rehabilitation and diaphragm recovery after cervical injury and transplantation of olfactory ensheathing cells. *Neurobiology of Disease*.

[154] Gauthier P., Réga P., Lammari-Barreault N., Polentes J. (2002). Functional reconnections established by central respiratory neurons regenerating axons into a nerve graft bridging the respiratory centers to the cervical spinal cord. *Journal of Neuroscience Research*.

